# Living with the past: larval eastern oyster (*Crassostrea virginica*) culture salinity affects post-metamorphic physiological performance

**DOI:** 10.1093/conphys/coaf077

**Published:** 2025-11-07

**Authors:** Emily Fuqua, Sandra Brooke

**Affiliations:** Department of Biological Science, Florida State University, King Life Sciences Building, 319 Stadium Drive, Tallahassee, FL 32304, USA; Coastal and Marine Laboratory, Florida State University, 3618 US-98, St. Teresa, FL 32358, USA

**Keywords:** Carry-over effects, condition index, *Crassostrea* virginica, Eastern oyster, energy expenditure, respiration, restorative aquaculture

## Abstract

Anthropogenically induced environmental change has contributed to population declines of important estuarine species, such as oysters. Some restoration programs focused on severely depleted oyster populations in estuarine environments are using hatchery-sourced animals to supplement low wild recruitment. However, carry-over effects, when early life experiences affect later life responses, are known to affect the success of cultured individuals in the wild. The objective of this study was to investigate carry-over effects on eastern oyster (*Crassostrea virginica*) larvae cultured under a range of salinities—an important environmental stressor on natural populations. Eastern oyster larvae were grown and settled across a range of salinities until large enough to transplant onto two field sites with different average salinities. Larval culture salinity significantly affected post-metamorphosed oyster growth rates until 45 days post-set, where oysters from suboptimal low salinity cultures grew faster immediately post-metamorphosed. Later, larval culture salinity significantly affected oxygen consumption rates and condition index of oysters from the field, and field site significantly interacted with larval culture salinity on physiological metrics. High larval salinity cultures produced oysters with lower energetic expenditures and higher condition index values, on average. Long-term physiological performance of animals depended on both the early culture environment and the subsequent field conditions, and because of the interaction of culture conditions and transplant site, care should be taken to select culture conditions that match those at target relocation sites.

## Introduction

Coastal waters and estuaries are the interfaces between freshwater and marine systems, and bear the brunt of anthropogenically driven environmental change due to their proximity to human population centres (Lotze *et al.*, 2006; Halpern *et al.*, 2007, 2008). Historically highly productive ecosystems, estuaries are undergoing alterations in long-term water quality regimes and increases in extreme short-term events such as seasonal anoxic zones and marine heat waves (Correll, 1978; Wilson, 2002; Howarth *et al.*, 2011; Mazzini and Pianca, 2022; Tassone *et al.*, 2022). These changes have significantly altered biological communities, population dynamics, and biomass of critical foundational species (Lotze *et al.*, 2006; Beck *et al.*, 2011; Zu Ermgassen *et al.*, 2012).

The eastern oyster (*Crassostrea virginica)* is a foundational species in coastal and estuarine systems throughout its range along the Western Atlantic. Eastern oysters create complex biogenic reefs, which are sustained by cycles of larval recruitment onto conspecifics’ shells (Crisp, 1967; Poirier *et al.*, 2019). This species contributes many ecological services, including providing essential habitat for other species, shore and erosion protection, and filtration of the water (Tolley and Volety, 2005; Scyphers *et al.*, 2011; Hoellein *et al.*, 2015). The eastern oyster is also an important fishery species and has a long history of exploitation throughout its range (Kirby, 2004). Due to their proximity to local human population centres, anthropogenic change has contributed to major eastern oyster population declines, particularly the Chesapeake Bay in the 1900s, and more recently, Apalachicola Bay, Florida in 2012 (Kirby, 2004; Mackenzie, 2007; Pine III *et al.*, 2015; Schulte, 2017). Consequently, restoration efforts for eastern oysters in the United States are now common.

Oyster restoration primarily focuses on subsidizing hard substrate for larval settlement (Powell *et al.*, 2006; Bersoza Hernández *et al.*, 2018). In systems with insufficient larval supply, restoration aquaculture strategies are used to supplement wild recruitment, which uses oysters sourced from hatcheries, remote-setting facilities, and community-based “oyster gardening” programs (Rossi-Snook *et al.*, 2010; Kennedy *et al.*, 2011; Steppe *et al.*, 2016). Hatcheries rear larval oysters in stable conditions to maximize larval survival, and larvae are settled on substrate in the hatchery or in a remote-setting facility. Then young oysters are placed in the target restoration areas (Rossi-Snook *et al.*, 2010; Steppe *et al.*, 2016; Wasson *et al.*, 2020). As coastal areas are facing environmental change, the success of restorative aquaculture will require that cultured animals are resilient to environmental conditions after deployment into natural systems.

In many taxa, the environment experienced by early life stages affects responses in later developmental stages; these phenomena are known as carry-over effects (Lindström, 1999;Jackson and Brown, 2011; Scherer and Smee, 2017; Donelan *et al.*, 2021). Carry-over effects can impact responses such as behaviour, physiology, morphology, and survival (Jackson and Brown, 2011; Scherer and Smee, 2017; Donelan *et al.*, 2021). For example, lack of predators in early culture of Atlantic salmon (*Salmo salar*) lead to fingerling fish behaving differently in the presence of predators in natural conditions and can translate into increased mortality due to predation (Jackson and Brown, 2011; Solberg *et al.*, 2015). In marine bivalves, such as blue mussels (*Mytilus edulis*) and eastern oysters, lack of predator cues cause mussels and oysters to allocate energy to growth instead of shell strength, which also increases mortality rates in natural conditions due to predation (Freeman and Byers, 2006; Scherer and Smee, 2017; Belgrad *et al.*, 2021).

Carry-over effects on physiology and stress responses generally work as silver-spoon effects, when animals grown in optimal environments perform best later (Lindström, 1999; Hettinger *et al.*, 2013) or as pre-exposure effects, when animals pre-exposed to suboptimal conditions perform best in later environments (Encomio and Chu, 2007; Pereira *et al.*, 2020; Donelan *et al.*, 2021). For example, Olympia oyster (*Ostrea lurida*) larvae grown in lower pCO_2_ conditions (i.e. less stressful) have higher survival and growth rates as post-settled juveniles planted into different field conditions when compared to larvae grown in higher pCO_2_, illustrating a silver-spoon effect (Hettinger *et al.*, 2013). Alternatively, Encomio and Chu (2007) found that eastern oysters subjected to sublethal heat shocks have a higher survival to later high heat exposures, which illustrates pre-exposure effects. Similar results have been reported in other oyster species, such as the flat oyster (*Ostrea angasi*) and the Sydney rock oyster (*Saccostrea glomerata*; Pereira *et al.*, 2020); however, pre-exposure to stressors can lead to ambiguous and even detrimental results. Donelan *et al.* (2021) exposed eastern oyster oysters to combinations of stressors (i.e. hypoxia and elevated temperatures), but the only treatments that later showed significantly higher performance were those initially exposed to the stressors separately and then re-exposed to the stressors concurrently. Oysters exposed to concurrent stressors initially and later performed significantly worse than other stressor-exposed treatments (Donelan *et al.*, 2021). These studies suggest, while early physical environment, such as salinity, pH, or temperature, do have lasting effects on later performance, these effects are context specific, depend on the later environment, and can be beneficial or detrimental to animal performance (Encomio and Chu, 2007; Hettinger *et al.*, 2013; Donelan *et al.*, 2021).

As estuaries are known as highly variable environments, understanding how early life environments affects later performance will be critical to producing restoration animals that are resilient to environmental change and will be important for the success of restoration programs. The objective of this study was to determine how culturing eastern oyster larvae in different salinities affected their physiological performance post-metamorphosis and when transplanted into different field environments. Post-metamorphosed oysters that were pre-exposed to a range of sub-optimal salinities as larvae were expected to perform best under field conditions due to the pre-exposure carry over effects that have been documented in this species previously (Encomio and Chu, 2007; Donelan *et al.*, 2021). Salinity was chosen as the focal water quality parameter, because it is known to affect oyster growth and physiology throughout its life cycle (Casas *et al.*, 2018; Scharping *et al.*, 2019; McFarland *et al.*, 2022) and it is highly variable within and between estuaries. Additionally, the salinity in which larvae are cultured will vary among hatcheries based on location. Since oysters are sessile, they cannot escape stressful conditions, and physiological performance is critical to the animal’s survival. Physiological factors such as respiration and condition index were used as performance metrics to determine how early life environment affects oyster performance later in life.

## Materials and Methods

### Ethical declarations

All broodstock collection was permitted under Florida Fish and Wildlife Special Activity Licence #22-2197A-SR.

### Collection, spawning, and larval care

All larval culture and laboratory animal care took place in the Florida State Coastal and Marine Lab (FSUCML) shellfish hatchery in St. Teresa, Florida, USA. Wild eastern oysters with mature gonad were collected from Alligator Harbour, FL by gently breaking the oysters off subtidal substrate. Oysters were scrubbed with wire brushes and freshwater, and all shell-associated epibionts were removed. On 14 September 2023, 33 oysters (13 males and 20 females) were opened and stripped spawned according to Fuqua and Brooke (2025). Once successful fertilization (i.e. appearance of polar bodies) was observed under a microscope, eggs were split evenly and poured into two 1100 L tanks filled with 1 μm filtered seawater at a salinity of 25 and a temperature of 27°C. After 24 h, tanks were drained, and larval oysters were retained on a 40-μm sieve. Larvae were subsampled to estimate total number and then split evenly into ten 170 L conical-bottom tanks filled with 1 μm filtered seawater at a salinity of 25 and a temperature of 27°C. All tanks were stocked with an estimated 1.3 million larvae tank^−1^ (8 larvae ml^−1^), and the tanks were randomly assigned to one of ten treatment salinities (10, 12, 14, 16, 18, 20, 22, 24, 26 and 28). Salinity was adjusted from the initial 25 at a rate of 3–4 salinity units hour^−1^ (no more than 10 units total day^−1^), by adding either reverse-osmosis (RO) water to lower salinity or 1 μm filtered ambient seawater (28–30 salinity) to increase it. These rates were based on mean daily rates of salinity changes calculated from local environmental data and were meant to represent rates of change larvae would be exposed to under wild conditions (NOAA National Estuarine Research Reserve System, 2023). All larvae were at treatment salinities within 48 hours.

Daily water quality (salinity, temperature and pH) was recorded using digital salinity (Hanna Instruments) and pH-temperature (BlueLab) probes, and larvae received water changes. Water changes were carried out as detailed in Fuqua and Brooke (2025). Larvae were fed mixtures of live microalgae daily, and all tanks received the same ratios and concentration of microalgal species daily (Table S1). All tanks were aerated continuously. Salinity was kept within 0.5 of treatment salinities, and temperature averaged 26.3°C (±0.5) during the larval period. Tank pH averaged 7.91 (±0.13) during the larval stage. Every second day, subsamples of live larvae were counted to estimate survival. Photos of the subsamples of live larvae (minimum of 25 larvae treatment^−1^) were taken for growth measurements.

Once larvae showed signs of settling competency, (i.e. the presence of eyespots and feet), competent larvae were removed from tanks daily, poured through a coffee filter, gently folded into a damp towel, and saved live in a refrigerator set at 10°C. Non-competent larvae were restocked in tanks and allowed to continue developing. After 72 h, total number of competent larvae was estimated, and all competent larvae were transferred into settling tanks.

### Oyster settlement and early juvenile care

Settling tanks were separate 15-L static buckets equipped with an air stone and settling material (150 × 150 mm Mylar sheet, 150 × 150 mm high-density polyethelene sheet, and cultch). Tanks were filled with seawater matching the larval treatment salinity, competent larvae were added, and tanks were covered to provide a dark environment to encourage metamorphosis. Larvae were allowed 72 h to settle on materials provided. Water was changed, tanks were cleaned, and animals were fed daily a live algal mixture (Table S1). After 72 h, any larvae that failed to settle were removed, and the water in tanks was slowly brought to ambient salinity to establish flow-through.

Once all treatments underwent settlement, the juveniles were housed by treatment in separate 15 L upwelling, flow-through tanks. Tanks were provided with 50-μm filtered seawater for 18 h each day, but flow was shutoff for the remaining 6 h when microalgae was added to allow the oysters to feed. Oysters were fed a live algal mixture daily (Table S1). Water quality was recorded daily from the incoming water line that fed flow-through tanks, and all tanks and post-metamorphosed oysters were rinsed daily with freshwater to remove waste build-up. Photos of post-settled oysters were taken on days 47 and 66 post-spawn for growth measurements.

### Field grow-out

On 20 November 2023, juveniles from each treatment were taken to field grow-out sites on open-water leases maintained by the FSUCML staff in Alligator Harbour (29° 55′ 22″ N 84° 24′ 57″ W) and Oyster Bay (30° 03′ 25″ N, 84° 20′ 19″ W), Florida. Randomly subsampled oysters were placed in 0.75-mm mesh bags at an estimated density of 2500 oysters bag^−1^ (1 mesh bag treatment^−1^ lease^−1^), and each mesh bag was secured into a separate 10-mm floating aquaculture cage. Aquaculture cages were secured onto floating lease lines in a randomized order. Every 14 to 21 days, field sites were visited. Bags were cleaned and replaced if damaged, and a haphazard subsample of oysters (minimum of 25 treatment^−1^) were selected for growth photos. To monitor food availability at grow-out sites, each time bags were checked, three water samples were taken at each grow-out site. At the lab, water samples were gently inverted 3–5 times to homogenize sample, and three 10 μl subsamples were pipetted onto haemocytometer slides. Live algal counts were estimated from each subsample using an Invitrogen Countess II FL Automatic Cell Counter equipped with an EVOS Cy5 2.0 Light Cube (ThermoFisher Scientific). Counts were averaged by water sample and then by site to calculate algae density present at site (Table S2). Water quality at field sites was monitored using the YSI EX02 (Xylem Analytics) water quality monitors in Alligator Harbour and Oyster Bay (Table S2). Oysters were grown at field sites for 77 days and retrieved on 7 February 2024. Oysters were brought back to the FSUCML and cleaned, and final growth pictures were taken.

### Growth measurements

Photos of larval oysters and post-metamorphosed oysters smaller than 1 mm were taken using a 1 × 1 mm gridded Sedgewick slide and a compound microscope on 4× magnification. Photos of post-metamorphosed oysters larger than 1 mm were taken by placing animals in a clear petri dish with a marked ruler in the photo. ImageJ software was used to take measurements from a minimum of 25 individuals treatment^−1^ for each set of photos. For larval oysters, shell height (length of the shell from the centre of the hinge to the centre of the perpendicular edge of the shell) was recorded, and for post-metamorphosed oysters, shell height and shell width (length of the perpendicular axis to shell height) were recorded.

### Respirometry

Respiration measurements were conducted on 4–6 oysters treatment^−1^, haphazardly chosen from those brought back from field sites. Respirometry measurements began four days after field retrieval. During this time and while respirometry trials were ongoing, oysters were kept separate by treatments and maintained in 2.5 L flow-through tanks supplied with ambient seawater that was UV-sterilized and filtered to 20 μm. Water quality was recorded daily. All tanks were fed 150 000 cells ml^−1^ of live algae daily until 24 h prior to respirometry measurements. Oysters were starved for 24 h before respirometry to limit the influence of postprandial metabolism on oxygen consumption. Order of respirometry measurements was randomized among treatments. Oysters were scrubbed immediately prior to respirometry measurements to remove epibionts, then placed in sealed 60-ml glass containers with a magnetic mixing bar. Seawater for respiration measurements was UV-sterilized seawater filtered to 0.35 μm at ambient water quality (temperature: 21°C ± 1, salinity 30 ± 1). Oxygen concentration in the sealed chamber was measured with a PreSens SP-PSt3-NAU sensor and fibre optic contactless probe. Each oyster was allowed to acclimate in the chamber for as long as necessary, and no recordings were taken until the valves had been continuously open for 30 minutes. Oxygen concentration of the seawater was recorded while the valves were visibly open and the chamber was sealed shut. Recordings lasted for at least 10 min animal^−1^. After the oxygen consumption measurements had been taken, volumetric displacement of each animal was measured, then, the animal was shucked and tissues were removed. The shell was placed back into the chamber, and oxygen concentration was measured in the sealed container for 5 min. This measurement was used as the background respiration measurement for the animal. Immediately after removal from the shell, tissue wet weight was measured, and the shell and tissues were frozen at −20°C until processing for condition index and ash-free dry mass. After respirometry trials were finished, frozen samples were thawed. Tissues and shells were dried at 60°C for 24 h, and dry weights were recorded. Tissues were then transferred to a muffle furnace at 550°C for 4 h, ash weight was recorded, and ash-free dry mass was calculated. Condition index was calculated from dry shell weights and ash free dry mass measurements (Davenport and Chen, 1987).

### Data analysis

All data analysis occurred in RStudio (2023.06.0 + 421; R version 4.2.2), and data were analysed using generalized linear models (glms). Larval culture salinity was used as a fixed effect in all analyses, and outplant site was used as a fixed effect in analyses of oyster growth rate during field grow out, oxygen consumption rate, and condition index. Significant interactions between larval culture salinity and outplant site were tested for in these models as well (Table S3).

Larval proportion survival was calculated as the estimated number of live larvae at the end of the larval culture period divided by estimated number of live larvae at the beginning. Larval growth rate was calculated from photo growth measurements as the average shell height of the larvae in the treatment at the end of culture minus the average shell height of larvae at the beginning of culture divided by the number of culture days (mm day^−1^). Proportion of larvae to reach competency was calculated as the estimated number of competent larvae in a treatment divided by number of live larvae at the beginning of larval culture. Post-metamorphic oyster growth rates during the different periods of time (i.e. growth in hatchery and growth in the field) were calculated at the average shell height of the oysters in the treatment at the beginning of the period subtracted from the average shell height of the oysters in the treatment at the end of the period divided by the number of days between beginning and end of period. Oxygen consumption rate was calculated as the rate of decrease in oxygen concentration during the live animal measurement minus the rate of decrease in oxygen concentration of the shell and water with no oyster tissue present. Oxygen consumption rates for each animal were standardized by the ash-free dry mass of tissues. The condition index of animals was calculated as recommended by Davenport and Chen (1987) for tissue samples that are frozen prior to processing, which was ash-free dry mass of tissue divided by dry shell weight multiplied by one hundred.

For larval proportion survival and larval proportion competency, glms with binomial error distributions (logit link functions) were fit with and without second-degree polynomials terms. For the remaining response variables, glms with Gamma error distributions (log link functions) were fit with and without second-degree polynomial terms. For all glms tested, residuals from models were inspected using simulated residuals from the “DHARMa” package. Simulated residuals were tested with Kolgormorov–Smirnov uniformity tests, dispersion tests, and outlier tests to evaluate model fit and test model assumptions. Additionally, AIC values were compared for glms with and without polynomial terms. For each response variable, the best-fit model selected was the glm that passed all residual tests and had the lowest AIC value of the glms tested (Table S3). For hypothesis testing, glms were tested against null models and deviance values (G) were used to determine significance of fixed effects and interactions between fixed effects with a cut off of *P* = 0.05.

Water quality of larval salinity treatments was tested for differences in temperature and pH using one-way ANOVAs. Water quality of field sites during grow-out was obtained from FSUCML HydroSphere (FSUCML HydroSphere Water Quality Monitoring System, 2024). Daily mean of all water quality parameters during field grow-out was calculated, and sites were paired by date. Paired *t*-tests were used to compare water quality of the two field sites (Table S2). Algae density from algal samples collected at field sites was also compared using a paired *t*-test (Table S2).

## Results

### Effect of larval culture salinity on larval performance

**Figure 1:**
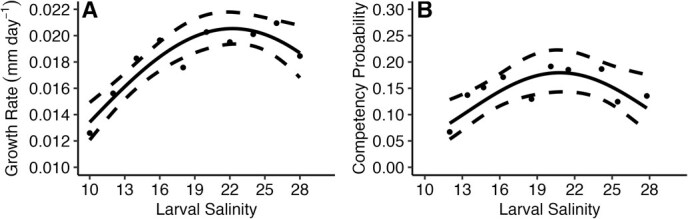
Effect of larval culture salinity on (**A**; left) larval growth rate (mm day^−1^) and (**B**; right) probability of larval competency. Points represent mean treatment larval growth rates (**A**) and mean treatment proportion of competent larvae. Solid lines represent best-fit glms, and dashed lines represent the 95% confidence intervals of the models.

Larval culture salinity had a significant relationship with larval survival (*G*_2_ = −36.4, *P* < 0.001), larval growth rate (*G*_2_ = −0.165, *P* < 0.001; Fig. 1A), and proportion of larvae that became competent to settle (*G*_2_ = −8.77, *P* < 0.001; Fig. 1B). Larval survival and competency peaked between 15 and 20 and decreased in extreme salinities (Fig. 1B). While larval survival ranged from 0.40 to 0.83, proportion of larvae that reached competency ranged between 0.06 and 0.19 in larval salinity treatments (Fig. 1B). Larval growth rate increased with salinity from 0.013 mm day^−1^ 10 to 0.02 mm day^−1^ at 20 and remained high with increasing salinity (Fig. 1A). Larvae in higher salinities grew 0.6 times faster than those cultured at the lowest salinities. In treatment tanks, neither temperature nor pH differed among larval salinity treatments (temperature: *F*_11_ = 0.75, *P* = 0.69; pH: *F*_11_ = 1.41, *P* = 0.17).

### Effect of larval culture salinity on post-metamorphic growth rate

**Figure 2:**
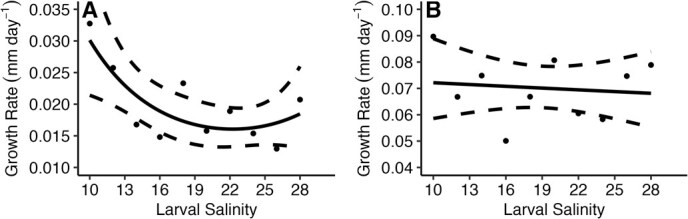
Effect of larval culture salinity on (**A**; left) post-metamorphosed oyster growth rate (mm day^−1^) between 14 and 44 days post-fertilization and (**B**; right) post-metamorphosed oyster growth rate (mm day^−1^) between 45 and 66 days post-fertilization. Points represent mean treatment growth rates. Solid lines represent best-fit glms, and dashed lines represent the 95% confidence intervals of the models.

**Figure 3:**
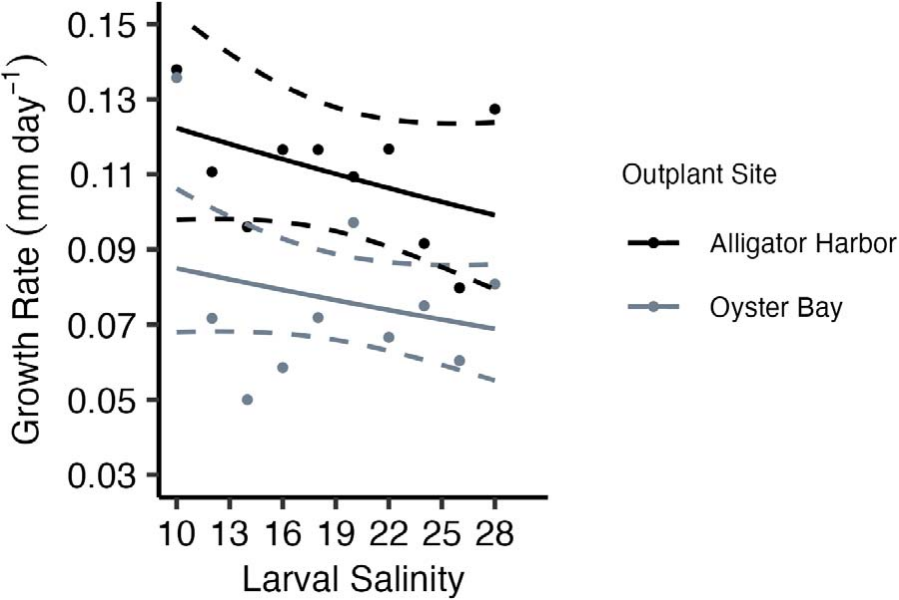
Effect of larval culture salinity on post-metamorphosed oyster growth rate (mm day^−1^) after being deployed at two field sites (Alligator Harbour in black, and Oyster Bay in grey) for three months. Points represent average growth rates of oysters in each treatment. Solid line represents best-fit glm, and dashed lines are the calculated 95% confidence interval of the model.

Post-metamorphosis, larval culture salinity significantly affected growth rate during the first growth sampling period in a common environment, up to 47 days post-metamorphosis (*G*_2_ = −0.446, *P* = 0.009; Fig. 2A). During this period, oysters cultured in low larval salinities grew twice as fast as the oysters cultured in mid- to high larval salinities (Fig. 2A). However, after 47 days post-metamorphosis in a common environment, larval culture salinity no longer significantly affected oyster growth rate (*G*_1_ = −0.0036, *P* = 0.73; Fig. 2B). This trend continued through the field grow-out period, and larval culture salinity did not significantly affect oyster growth rates in the field (*G*_1_ = −0.097, *P* = 0.19; Fig. 3). Oyster growth rates in the field were significantly affected by site (*G*_1_ = −0.66, *P* = 0.0005; Fig. 3), but no significant interaction between larval culture salinity and outplant site was found (*G*_1_ = −0.004, *P* = 0.77). Mean growth rate of oysters in Alligator Harbour during field grow out was 0.11 mm day^−1^ (± 0.02), which was 0.7 times faster than the mean growth rate of oysters in Oyster Bay, 0.08 mm day^−1^ (± 0.02; Fig. 3).

### Effect of larval culture salinity on respiration and condition index

**Figure 4:**
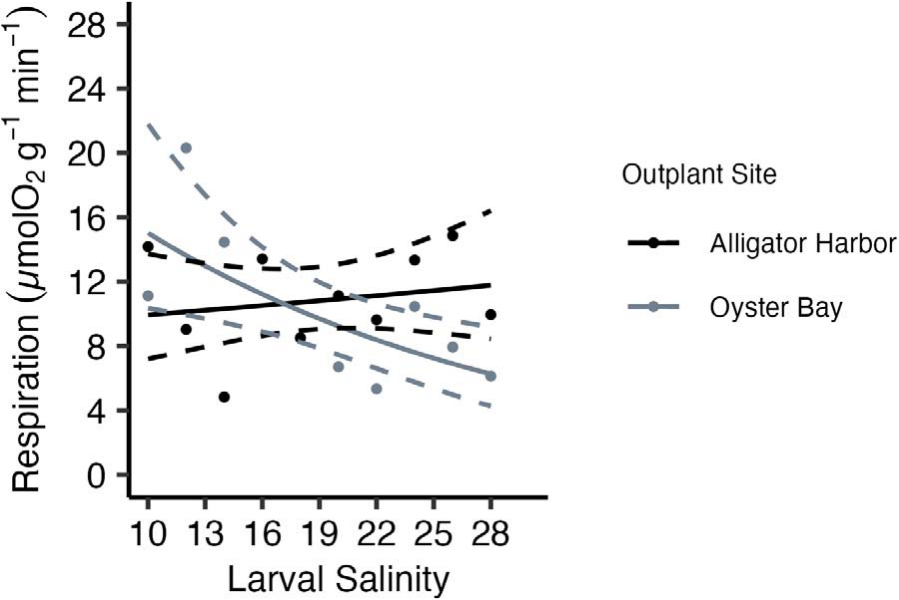
Effect of larval culture salinity and field site on oyster respiration rate (μmolO_2_ g^−1^ min^−1^) of post-metamorphosed oysters. (Alligator Harbour in black, and Oyster Bay in grey). Points represent mean treatment oxygen consumption rates. Solid line represents best-fit glm, and dashed lines are the calculated 95% confidence interval of the model.

After the field grow out, larval culture salinity did have a significant effect on oxygen consumption rates of oysters (*G*_3_ = −3.77, *P* = 0.036; Fig. 4). Field site also significantly affected oxygen consumption rates (*G*_2_ = 3.07, *P* = 0.031; Fig. 4), and a significant interaction was present between larval culture salinity and outplant site (*G*_1_ = −2.89, *P* = 0.011; Fig. 4). Within the oysters planted in Oyster Bay, oxygen consumption rates were approximately three times higher in oysters cultured at low larval salinities, and oxygen consumption rates decreased from 11.1 μmol g^−1^ min^−1^ (±6.2) to 6.12 μmol g^−1^ min^−1^ (±4.2) as larval culture salinity increased (Fig. 4). Alternatively, within the group of oysters planted in Alligator Harbour, oxygen consumption rates remained consistent across larval culture salinity and averaged 10.9 μmol g^−1^ min^−1^ (±3.1; Fig. 4).

**Figure 5:**
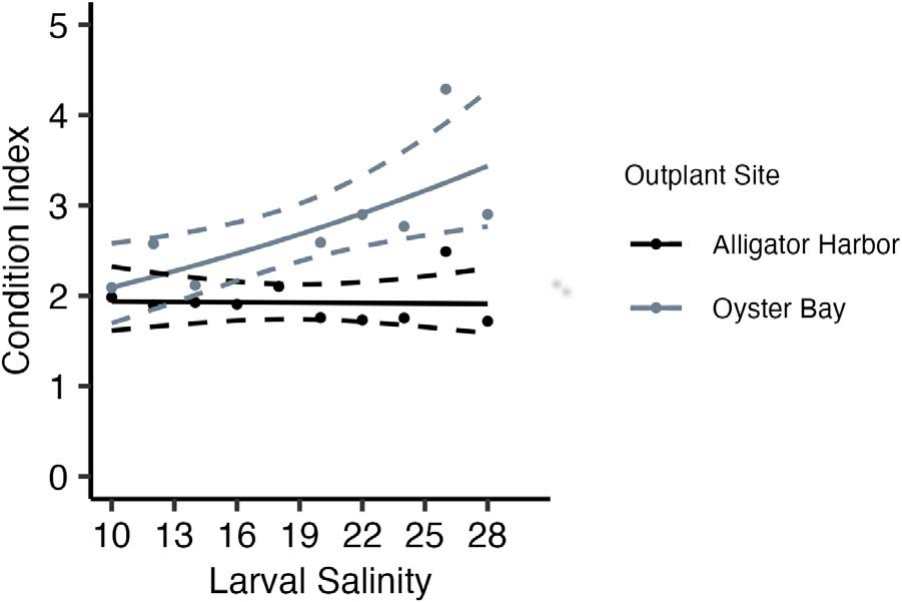
Effect of larval culture salinity and field site on oyster condition index. Colours indicate different field sites (Alligator Harbour in black, and Oyster Bay in grey). Points represent mean treatment condition. Solid line represents best-fit glm, and dashed lines are the calculated 95% confidence interval of the model.

Condition index was also significantly affected by larval culture salinity (*G*_3_ = −3.86, *P* < 0.001; Fig. 5) and outplant site (*G*_2_ = 3.21, *P* < 0.001; Fig. 5), with a significant interaction between the two fixed effects (*G*_1_ = −0.67, *P* = 0.029; Fig. 5). The oysters planted in Oyster Bay were more affected than those planted in Alligator Harbour. The condition index of oysters planted in Oyster Bay averaged 2.09 (±0.4) in oysters cultured as larvae in low salinities and increased as high as 4.29 (±0.7) as larval culture salinity increased (Fig. 5). However, the oysters planted in Alligator Harbour had similar mean condition index of 1.93 (±0.2) across the larval culture salinities (Fig. 5).

## Discussion

With estuaries undergoing environmental change, resiliency of the animals being used for restoration will be critical to the success of the restoration programs. This study showed that the salinity environment larval eastern oysters are cultured in affects their physiological responses immediately as larvae, as recent post-metamorphosed oysters, and up to four months after transplant into the natural environment. Additionally, this study demonstrated that later life environment is important in determining how carry-over effects manifest due to the interaction between larval culture salinity and outplant sites (Figs 4 and 5). For this reason, the larval environment that promotes larval growth and development may not produce the most resilient oysters in estuarine environments. Additionally, similar to other carry-over effects, the effects found in this study could have implications for organismal performance and resilience under natural conditions. Collectively, these results indicate that consideration of hatchery and planting environments could influence the success of oyster restoration programs.

While in culture, hatcheries focus on maximizing larval production numbers (e.g. larval growth and survival), meaning these animals are grown in stable, low stress conditions. Since the goal of aquaculture is to grow an organism to a sellable size as quickly as possible, hatchery techniques are optimized according to this goal. This type of environment could give rise to silver-spoon effects, where larvae grown in low stress environments perform better across later environments (Lindström, 1999; Hettinger *et al.*, 2013). However, this study did not show evidence of silver-spoon effects. Optimal larval treatments—mid-range salinities that maximized growth, development to competency, and survival in the larval stage—displayed the lowest growth rates in early post-metamorphosed oysters (Figs 1 and 2) and did not perform significantly better than oysters cultured as larvae in sub-optimal conditions in field environments. Instead, oysters cultured in sub-optimal low salinity displayed the highest growth rate as early post-metamorphosed oysters (Fig. 2). This pattern may be due to the sub-optimal salinity environments and hatchery methodology selecting for individuals with fast growth and development rates in these larval treatments, and the patterns in larval survival and proportion of competent larvae across salinity treatments indicate some support for this hypothesis (Fig. 1). Another mechanism for the increase in post-metamorphic growth rate is compensatory growth, where individuals from sub-optimal environments grow faster to overcome early life setbacks, and this mechanism is documented in marine bivalves, including the eastern oyster (Gobler and Talmage, 2012; Keppel *et al.*, 2016). Regardless of the acting mechanism producing these changes, these results indicate using standard aquaculture techniques to culture animals may not lead to the best outcome for restoration programs.

In some cases, pre-exposing animals to stress can lead to carry-over effects that increase resiliency to stress later, such as in the case of sub-lethal heat shocks prior to lethal heat shocks (Encomio and Chu, 2007; Pereira *et al.*, 2020), although results can be ambiguous (Donelan *et al.*, 2021). While the present study did not specifically test if matching larval conditions to specific field site environments increased performance, this study demonstrated that later environment is critical in determining how carry-over effects manifest in animals. Even though later oyster growth patterns were only significantly affected by outplant site, underlying patterns in energy expenditure and condition index were still being significantly driven by larval culture environments from four months prior, which interacted significantly with outplant site (Figs 4 and 5). In restoration, early life culture conditions that may be optimal for oysters in some sites may have adverse effects for oysters being planted in other sites. Although culturing groups of animals for specific sites may not be feasible, this information could lead to metrics of site suitability when choosing which sites within recruitment-limited and depleted estuaries could be restored using hatchery-sourced oysters.

In many oyster restoration programs, post-metamorphosed oysters are transferred to field sites within months of settlement. In the present study, the largest carry-over effect on growth performance occurred within the first two months of settlement, corresponding with the standard timing of field transplant. Oysters cultured as larvae in low salinities displayed growth rates roughly twice as fast as oysters cultured as larvae in mid-range and high salinities (Fig. 4). In young oysters, size and growth rate can be an important mediator in biological interactions such as predation and competition (Eggleston, 1990; Krassoi *et al.*, 2008; Pusack *et al.*, 2018). Two major predators of the eastern oysters in the lower latitudes of this species’ range, blue crabs (*Callinectes sapidus*) and the southern oyster drills (*Stramonita haemastoma*), display size-specific preferences for oyster prey (Eggleston, 1990; Pusack *et al.*, 2018), which means the ability to grow faster and reach a size refuge may be a large determinant in success of restoration animals in predator-dense areas. Additionally, growth rates can mediate success in space competition, especially in the subtidal and lower intertidal zones (Krassoi *et al.*, 2008). Lower growth rates may reduce the success of restoration oysters competing for free space in natural environments, which will decrease the success of the overall restoration efforts. For oysters being transplanted for restoration, changes in growth rate could translate into differences in survival, performance and success in natural conditions.

Resiliency to environmental stress will increase survival of organisms in environments that change rapidly, such as estuaries (Knowles, 2002; Montagna *et al.*, 2002). For sessile animals, like oysters, physiological performance will be key to tolerating environmental variation. Understanding how physical environments affect energy expenditure and energy budgets of sessile animals is important to evaluating whether restoration aquaculture techniques will work for these species. In this study, the oysters planted at the Oyster Bay field site had lower growth rates than those planted in Alligator Harbour (Fig. 3). However, condition index showed the opposite pattern, and the Oyster Bay oysters had higher condition index values than Alligator Harbour (Fig. 5). This may indicate oysters planted in Alligator Harbour were investing more energy in growth, driving up growth rates and decreasing condition index, and oysters in Oyster Bay were diverting more energy to long-term storage, decreasing growth rate and increasing condition index. Within Oyster Bay, the oysters with highest energy expenditure corresponded with the oysters with the lowest condition index values, which are the oysters cultured at the lowest larval salinities, and as larval culture salinity increases, condition index values increase and energy expenditure decreases (Figs 4 and 5). In this study, the oysters are investing energy and drawing on energy stores differently according to the salinity experienced as larvae. Most likely, oysters with high condition indexes (i.e. large energy stores) will persist longer in times of stress due to the ability to pull on these energetic stores. Additionally, having lower standard energy expenditure will assist in saving those energy stores. In Oyster Bay, the oysters cultured at the highest larval salinities, which have low-energy expenditure rates and high condition index values, should be the most resilient animals to periods of environmental stress. In comparison, oysters planted in Alligator Harbour had relatively similar energy expenditures and condition index values, regardless of larval culture salinity. In this latter environment, resiliency of oysters may not be differentially affected by larval culture environment. In these cultured oysters, larval salinity does seem to affect long-term energy budgets of oysters depending on their later environment, which may impact animals being placed in wild, estuarine conditions.

The differences between physiological metrics between sites could be driven partially by transgenerational affects, such as transgenerational plasticity or local adaptation, as the broodstock for this study were from Alligator Harbour. Griffiths *et al.* (2021) found no evidence for transgenerational plasticity, a non-genetic mechanism for parents to influence offspring responses, in *C. virginica* oysters from Louisiana, and genetic families conditioned in low salinity sites did not produce larvae that performed better in low salinity challenges relative to the same genetic families conditioned in higher salinity sites (Griffiths *et al.*, 2021). Alternatively, local adaptation in salinity response has been documented in oyster species (Eireman and Hare, 2013; Bible and Sanford, 2016). Eireman and Hare (2013) report differences in offspring salinity response from *C. virginica* oysters collected along a salinity gradient in Delaware Bay, New Jersey. Parents were conditioned in common gardens, and parental origin significantly interacted with larval salinity treatment to affect larval survival (Eireman and Hare, 2013). In the present study, it is possible that local adaptation factored into the increase in growth rate of juvenile oysters in Alligator Harbour and influenced other physiological metrics. Further studies will be needed to understand how genetic factors may influence and interact with plastic carry-over effects to shape energetic budgets, physiological responses and resiliency of sessile marine organisms, such as oysters.

Ongoing restoration using aquaculture techniques for depleted and recruitment-limited oyster populations will continue to face the major environmental changes that estuarine and coastal systems are undergoing (Halpern *et al.*, 2008; Hale *et al.*, 2016; Tassone *et al.*, 2022). The animals being transplanted into these systems will need to be resilient to large-scale natural and anthropogenic environmental variation. However, using standard aquaculture practices may not lead to oysters that will be resilient in natural conditions due to differing end goals of aquaculture and restoration and the ecological impacts of culture-induced carry-over effects. To increase the efficacy of these restoration programs, practitioners may need to consider how the culture environment and field environment interact to affect organismal performance and long-term success or optimize culture techniques that increase long-term oyster performance.

## Supplementary Material

Web_Material_coaf077

## Data Availability

Data generated during this study are available on the figshare repository. DOI: https://doi.org/10.6084/m9.figshare.30401323.v1
