# Supplementary material for: Living with the past: larval eastern oyster (Crassostrea virginica) culture salinity affects post-metamorphic physiological performance
Source: Conserv Physiol. 2025 Nov 7;13(1):coaf077. doi: 10.1093/conphys/coaf077 (PMC12596496; doi:10.1093/conphys/coaf077)
Supplement: Web_Material_coaf077 [file web_material_coaf077.pdf]

Supplemental Materials

Title: Living with the past: larval eastern oyster (*Crassostrea virginica*) culture salinity affects post-metamorphic physiological performance

Authors: Emily Fuqua[1], Sandra Brooke[2]
Affiliations:
[1]Florida State University Department of Biological Science, King Life Sciences Building, 319 Stadium Drive, Tallahassee, FL 32304
[2]Florida State University Coastal and Marine Laboratory, 3618 US-98 , St. Teresa, FL 32358

*Correspondence to: efuqua@fsu.edu

Table S1: Larval and post-metamorphosed oyster feeding schedule including days post spawn, algal species fed (* indicates a flagellate species and † indicates a diatom), and total concentration of feed added. Algal species composition was decided based on stage of larvae and which algal species were available to harvest.

Days post spawn	Algal Species	Total Concentration (cells mL^{-1})
1	*Tisochrysis lutea**	40,000
2	*Tisochrysis lutea**	50,000
3	*Tisochrysis lutea**	60,000
4	*Tisochrysis lutea**	65,000
5	*Tisochrysis lutea**	75,000
6	*Tisochrysis lutea**<br>*Chaetoceros gracilis†*	80,000
7	*Tisochrysis lutea**<br>*Chaetoceros gracilis†*<br>*Tetraselmis chui**	85,000
8	*Tisochrysis lutea**<br>*Chaetoceros gracilis†*<br>*Tetraselmis chui**	95,000
9	*Tisochrysis lutea**<br>*Chaetoceros gracilis†*<br>*Tetraselmis chui**<br>*Pavlova pingus**	105,000
10	*Tisochrysis lutea**<br>*Chaetoceros gracilis†*<br>*Tetraselmis chui**<br>*Pavlova pingus**	115,000
11	*Tisochrysis lutea**<br>*Chaetoceros gracilis†*<br>*Pavlova pingus**	120,000
12	*Tisochrysis lutea**<br>*Chaetoceros gracilis†*<br>*Tetraselmis chui**<br>*Pavlova pingus**	125,000
13	*Tisochrysis lutea**<br>*Chaetoceros gracilis*<br>*Tetraselmis chui**<br>*Pavlova pingus**	130,000
14	*Tisochrysis lutea**<br>*Chaetoceros gracilis†*<br>*Tetraselmis chui**<br>*Thalassiosira weissflogii†*	140,000
15	*Tisochrysis lutea**<br>*Chaetoceros gracilis†*<br>*Tetraselmis chui**<br>*Thalassiosira weissflogii†*	140,000

16	*Tisochrysis lutea**<br>*Chaetoceros gracilis†*<br>*Tetraselmis chui**<br>*Pavlova pingus**	140,000
17	*Tisochrysis lutea**<br>*Chaetoceros gracilis†*<br>*Tetraselmis chui**	140,000
18	*Tisochrysis lutea**<br>*Chaetoceros gracilis†*<br>*Tetraselmis chui**<br>*Thalassiosira weissflogii†*	140,000
19	*Tisochrysis lutea**<br>*Chaetoceros gracilis†*<br>*Tetraselmis chui**<br>*Thalassiosira weissflogii†*	140,000
20	*Tisochrysis lutea**<br>*Chaetoceros gracilis†*<br>*Tetraselmis chui**<br>*Thalassiosira weissflogii†*	140,000
21—26	*Tisochrysis lutea**<br>*Chaetoceros gracilis†*<br>*Tetraselmis chui**<br>*Thalassiosira weissflogii†*	150,000
27—40	*Tisochrysis lutea**<br>*Chaetoceros gracilis†*<br>*Tetraselmis chui**<br>*Thalassiosira weissflogii†*	200,000
41—57	*Tisochrysis lutea**<br>*Chaetoceros gracilis†*<br>*Tetraselmis chui**<br>*Thalassiosira weissflogii†*	250,000
58—66	*Tisochrysis lutea**<br>*Chaetoceros gracilis†*<br>*Tetraselmis chui**<br>*Thalassiosira weissflogii†*	300,000

Table S2: Water quality values measured at field grow out sites, including temperature (°C), salinity, pH, dissolved oxygen (DO; percent saturation), turbidity (Nephelometric Turbidity unit; NTU), and algal cell density (1000 cells mL^{-1}). Site values are given as mean (± standard deviation), and results of paired t-tests.

**Water Quality Parameter**	**Alligator Harbor Mean (± SD)**	**Oyster Bay Mean (± SD)**	**Significance test**
Temperature	14.5 (± 2.7)	14.1 (± 2.6)	$t_{64} = 6.38$, $P < 0.001$
Salinity	31.6 (±0.89)	21.3 (±5.1)	$t_{64} = 18.7$, $P < 0.001$
pH	8.02 (±0.05)	7.83 (±0.13)	$t_{64} = 12.1$, $P < 0.001$
DO	94.9 (±4.2)	93.4 (±4.3)	$t_{64} = 2.18$, $P = 0.03$
Turbidity	1.63 (±1.7)	2.05 (±1.9)	$t_{64} = -1.35$, $P = 0.18$
Algal Density	326 (±265)	334 (±349)	$t_{11} = -0.11$, $P = 0.92$

Table S3: Best fit generalized linear models for each response variable with its fixed effects, error distributions, and link functions (if applicable). * in fixed effects means model included a significant interaction between fixed effects of model.

**Response variable**	**Fixed effects**	**Error distribution**	**Link function**
Proportion larval survival	Larval culture salinity (2nd degree polynomial)	Binomial	Logit
Larval growth rate	Larval culture salinity (2nd degree polynomial)	Gamma	Log
Proportion competent larvae	Larval culture salinity (2nd degree polynomial)	Binomial	Logit
Early post-metamorphic growth rate	Larval culture salinity (2nd degree polynomial)	Gamma	Log
Late post-metamorphic growth rate	Larval culture salinity	Gamma	Log
Field growth rate	Larval culture salinity<br><br>Outplant Site	Gamma	Log
Oxygen consumption rate	Larval culture salinity<br>*<br>Outplant Site	Gamma	Log
Condition Index	Larval culture salinity<br>*<br>Outplant site	Gamma	Log
